# Psychiatric comorbid patterns in adults with attention-deficit hyperactivity disorder: Treatment effect and subtypes

**DOI:** 10.1371/journal.pone.0211873

**Published:** 2019-02-07

**Authors:** Fang-Ju Tsai, Wan-Ling Tseng, Li-Kuang Yang, Susan Shur-Fen Gau

**Affiliations:** 1 Department of Psychiatry, National Taiwan University and College of Medicine, Taipei, Taiwan; 2 Department of Psychiatry, En Chu Kong Hospital, New Taipei City, Taiwan; 3 Emotion and Development Branch, National Institute of Mental Heath, National Institutes of Health, Department of Health and Human Sevices, Bethesda, MD, United States of America; 4 Beitou Branch, Tri-Service General Hospital, National Medical Defense Center, Taipei, Taiwan; 5 Graduate Institute of Brain and Mind Sciences, College of Medicine, National Taiwan University, Taipei, Taiwan; 6 Graduate Institute of Clinical Medicine, College of Medicine, National Taiwan University, Taipei, Taiwan; QIMR Berghofer Medical Research Institute, AUSTRALIA

## Abstract

Psychiatric comorbidities are common in individuals with attention-deficit/hyperactivity disorder (ADHD). In this study, we sought to evaluate the effects of medication and childhood ADHD subtypes on psychiatric comorbidities among adults with ADHD as compared to healthy adult controls. We assessed 121 drug-naïve adults with ADHD, 93 treated adults with ADHD, and 145 healthy controls (age 18–36 years) using semi-structured psychiatric interviews, intelligence tests, and medical records. Drug-naïve adults with ADHD had more comorbidities than treated adults with ADHD and controls. Childhood ADHD-combined subtype, relative to ADHD-inattentive subtype, was associated with higher risks of comorbidities. Current medication treatment was associate with a higher risk for anxiety disorders, and longer treatment duration was associated with lower risks of mood disorders and sleep disorders. Our results indicate that no medication treatment, short treatment duration, and childhood ADHD-combined subtype are associated with increased risks for psychiatric comorbidities among adults with ADHD.

## Introduction

Symptoms of childhood attention-deficit/hyperactivity disorder (ADHD) often persist into adulthood among adults with this disorder [1]. The prevalence rate of adult ADHD is estimated at 2.5% based on a meta-analysis [2]. Although a few studies have reported psychiatric comorbidities in adults with ADHD [3, 4], much less is known about differential comorbid patterns in adults with ADHD who have received treatment vs. those who have not [5]. Similarly, data are very limited regarding psychiatric comorbidities in adults with different childhood ADHD subtypes [6, 7].

The existing literature has shown that adults with ADHD, relative to controls, are more likely to have psychiatric disorders [7, 8]. However, to date, only few studies have examined comprehensively the comorbid patterns within a single study [4, 8, 9]. In addition, some studies only focused on subgroups of adult ADHD such as males [5, 10] and those with major depressive disorder (MDD) [11].

Previous clinical trials have demonstrated the benefits and efficacy of medication in reducing clinical symptoms of ADHD [12] and improving quality of life [13], neuropsychological functions [14], and brain functions [15]. Furthermore, some clinical studies showed that a history of pharmacotherapy for ADHD decreased the risks for oppositional defiant disorder (ODD) [16], substance abuse [17–20], MDD [21], depression [22], or social phobia [23]. Methylphenidate was also found to be associated with causing [24, 25] or treating [24, 25] bulimia nervosa in case reports. However, other studies and case-reports revealed the opposite patterns, reporting that medication may be associated with an increased risk for substance use disorders [26], short-term nicotine use problems [27], and ODD/conduct disorder (CD) [28]. The directionality of these associations remained to be determined, as these relationships may be confounded by ADHD symptom severity and comorbid disorders such as CD [16]. Several follow-up studies show no evidence of stimulant treatment increasing or decreasing the risks for subsequent substance use disorders [29–32], anxiety [28], depression or MDD [28, 33] in children with ADHD when they enter adolescence and adulthood. Given the inconsistent findings regarding treatment effects on comorbidities and the limited research on the effect of treatment duration, more studies are needed.

Research on the associations between ADHD subtypes and psychiatric comorbidities in children and adolescents has suggested differential comorbid patterns [16] and brain functions [34] between ADHD-Combined type (ADHD-C) and ADHD-Inattentive type (ADHD-I), with more comorbid conditions reported in ADHD-C. For example, ADHD-C youths are more likely than ADHD-I youths to have ODD [35], CD [35–39], anxiety disorders [40], generalized anxiety disorder (GAD) [41], MDD [42], bipolar disorder [43, 44], substance use disorder [45, 46], sleep disorders/problems [47, 48], and eating disorders [49]. In contrast, ADHD-I youths have higher risks of anxiety disorders [36, 50] than ADHD-C youths. Adults with ADHD-C are more likely than adults with ADHD-I to have ODD [51], CD [6, 52, 53], panic disorder [52], specific phobia [52], mood disorders [52, 54], MDD [52], bipolar disorder [53], substance use disorders [6, 52, 54], and alcohol use disorder [6, 55]. Regarding sleep problems, sleepiness (greater sleep need) [56] and later circadian preference (evening orientation) [57] are associated with inattentive symptoms in adults with ADHD. However, few studies have investigated the association between childhood ADHD symptoms and adult psychiatric comorbidities [10, 58]. To address this gap, our study also aimed to investigate psychiatric comorbidities in adults with different childhood ADHD subtypes.

Taken together, little is known regarding the effects of medication, the duration of pharmacotherapy, and childhood ADHD subtypes on the psychiatric comorbid patterns in adults with ADHD. Hence, this study aimed to address these gaps in the literature by (1) examining group differences in psychiatric comorbid conditions among drug-naїve adults with ADHD, adults with ADHD treated with medication, and controls; (2) examining the effect of medication duration by comparing drug-naїve adults with ADHD, adults with ADHD treated longer than one year and those treated shorter than one year, and controls, in terms of psychiatric comorbid conditions; and (3) comparing adult psychiatric outcomes among adults with different childhood ADHD subtypes and controls.

## Materials and method

### Participants

The sample consisted of 214 adults with a clinical diagnosis of ADHD according to the DSM-IV diagnostic criteria and 145 healthy adult controls aged 18–36 years. Adults with ADHD were recruited from three sources: (1) a cohort of patients with ADHD assessed during their childhood [20]; (2) patients referred by psychiatrists from out-patient clinics or by family physicians in the National Taiwan University Hospital (NTUH), Taipei, Taiwan, and (3) new patients recruited via advertisements in the community. Our sample was part of a longitudinal follow-up study on ADHD from adolescence to young adulthood which was conducted from January 2011 to December 2015 [20]. The psychiatric interview was conducted from July 2011 to July 2015. To be consistent in the diagnostic criteria used in the diagnosis of ADHD and other psychiatric disorders, we used DSM-IV during the initial assessment (when DSM-5 was not yet available) and the follow-up assessment.

The new patients were screened using the Chinese version of the 6-item Adult ADHD Self-Report Scale-V1.1 [59, 60], followed by a telephone interview by a senior researcher with 10 years of experience in conducting clinical evaluations and psychiatric interviews with patients with ADHD and/or other psychiatric disorders. Potential cases with ADHD were invited for detailed clinical evaluations by the authors (SSG and LKY), followed by the 18-item Conners Adult ADHD Diagnostic Interview [61] by the corresponding author (SSG) for assessing childhood and current diagnosis of ADHD. All the participants were further interviewed by trained research psychologists using the modified adult ADHD supplement [62] of the Chinese version of the Schedule for Affective Disorder and Schizophrenia-Epidemiological Version (K-SADS-E) [63, 64] and SADS [13, 14] for assessing childhood and current diagnosis of ADHD and other psychiatric disorders, respectively. The psychometric property of the Chinese version of the K-SADS-E for DSM-IV was described in details in our previous work [63, 64] and summarized below.

Detailed information about the medication history for treating ADHD (including ever use, current use, duration of use, and daily dosage) and age onset of ADHD were included in the modified adult ADHD supplement of the Chinese K-SADS-E. Both ADHD participants and their parents received psychiatric interviews for the treatment history of medications mentioned above. The treatment data were validated by medical records in the NTUH if available; otherwise, they were validated by Taiwan’s National Health Insurance Claims data, which covers 99.9% of the population in Taiwan. Current use was defined as the presence of medication use for treating ADHD (methylphenidate or atomoxetine) in the past month regardless of the usage history prior to that. Ever use was defined as no medication use for treating ADHD in the past month but the use of these medications prior to the past month. Duration of use was defined as the total number of days on medications.

Adult healthy controls were recruited by advertisements on the internet, newspapers, and flyers at the colleges and public places based on the age and gender distributions of the ADHD group. They were screened by telephone interviews to rule out past or current ADHD symptoms, followed by clinical assessments and psychiatric diagnostic interviews using the Conners Adult ADHD Diagnostic Interview and the adult ADHD supplement of the Chinese K-SADS-E to ensure that they did not have lifetime or current diagnosis of ADHD. They also received the Chinese SADS for assessing other non-ADHD diagnoses.

All participants who had an IQ < 80 ascertained by the Wechsler Adult Intelligence Scale-Revised, had any serious medical illness such as cardiovascular disease, a history of learning disability, psychosis, pervasive development disorder or intellectual disability were excluded from the study.

### Procedure

The Research Ethics Committee of National Taiwan University Hospital (approval number, 2010003087R; ClinicalTrials.gov number, NCT01247610) approved this study before its implementation. After detailed explanations of the study, participants who provided written informed consent were recruited into the study. The final sample included 93 adults with ADHD who had been diagnosed with ADHD during childhood and treated for ADHD (treated ADHD), 121 adults with ADHD who have not been treated for ADHD (drug-naïve ADHD), and 145 adult controls without ADHD.

### Measures

#### The Chinese version of the K-SADS-E

The Chinese K-SADS-E for DSM-IV demonstrated good reliability and validity and had been widely used in a variety of clinical [65–68] and epidemiological [63, 69] studies in Taiwan. The corresponding author modified the ADHD, ODD and CD supplement of the Chinese K-SADS-E for the diagnoses of childhood ADHD, ODD and CD, and current ADHD diagnosis in adults [62, 70, 71]. Its original version, SADS [72, 73], in Chinese has been widely used in psychiatric research for assessing other DSM-IV non-ADHD psychiatric disorders [7, 13, 71].

#### The Chinese version of the Adult ADHD Self-Report Scale (ASRS)

The ASRS, an 18-item self-reported scale, was developed in conjunction with the revision of the World Health Organization (WHO) Composite International Diagnostic Interview (CIDI). The ASRS consists of two subscales, inattention (9 items) and hyperactivity-impulsivity (9 items), according to the 18 DSM-IV ADHD symptoms criteria. Each item asks how often a symptom occurred during the past six months on a 5-point Likert scale: 0, *never*; 1, *rarely*; 2, *sometimes*; 3, *often*; 4, *very often*. The psychometric property of the Chinese ASRS has been established in a sample of 1031 young males from an army base and 3298 young adults from two colleges in northern Taiwan [60]. It is widely used to measure ADHD symptoms in Taiwanese adults [56, 71, 74].

### Statistical analysis

Data were analyzed using SAS 9.1 (SAS Institute Inc., Cary, NC, USA). To examine sample characteristics among groups including age, gender, IQ, childhood and adulthood ADHD symptoms, and SES, we conducted chi-square tests (for categorical outcomes) and analyses of variance (ANOVA; for continuous outcomes). We first compared sample characteristics between 214 adults with ADHD (drug-naïve and treated) and 145 healthy controls, and then compared across 3 groups: (1) drug- naïve ADHD (n = 93); (2) treated ADHD (n = 121); and (3) healthy controls (n = 145) (Table 1). When significant group differences were found, post-hoc pairwise comparisons were conducted with corrections by the Scheffe test (Table 1). Descriptive results were displayed as frequencies, percentages, means and standard deviations.

**Table 1:** Sample characteristics.

mean±SD	ADHD (n = 214)			3.Controls (n = 145)	Difference between ADHD and controls		Difference between three groups		
	All ADHD	1.Drug-naïve ADHD (n = 121)	2.Treated ADHD (n = 93)		*F* or *χ*^2^	*p* value	*F* or *χ*^2^	*p* value	Post-hoc comparison
Male, n (%)	141 (65.89)	68 (56.20)	73 (78.49)	77 (53.10)	5.92	.015	16.88	< .001	
Age (18–36, in years)	24.76±5.16	26.86±4.72	22.02±4.39	23.78±4.21	3.64	.057	33.46	< .001	1>3>2
IQ profiles									
Full-scale IQ	108.09±10.61	109.85±10.25	105.80±10.69	111.04±9.93	6.90	.009	7.64	< .001	1,3>2
Verbal IQ	107.37±11.09	108.79±10.77	105.52±11.29	109.62±10.18	3.72	.055	4.36	.014	3>2
Performance IQ	108.24±12.09	110.12±12.06	105.81±11.75	111.54±10.98	6.74	.010	7.09	.001	1,3>2
Childhood ADHD symptoms									
Inattention	8.33±1.22	8.54±0.98	8.06±1.45	0.61±1.43	3008.58	< .001	1532.92	< .001	1>2>3
Hyperactivity-impulsivity	5.76±2.81	5.79±2.82	5.72±2.81	0.33±1.07	491.20	< .001	244.96	< .001	1,2>3
Adulthood ADHD symptoms									
Inattention	7.21±2.28	8.15±1.32	5.98±2.67	0.26±0.78	1243.76	< .001	828.04	< .001	1>2>3
Hyperactivity-impulsivity	3.86±2.78	4.40±2.79	3.17±2.62	0.12±0.43	258.51	< .001	144.05	< .001	1>2>3
Education in years	15.45±2.56	16.24±2.12	14.43±2.73	15.91±2.54	2.72	.100	15.55	< .001	1,3>2
Employment or school, n (%)									
Yes	186 (86.92)	105 (86.78)	81 (87.10)	134 (92.41)	2.70	.101	2.70	.259	
No	28 (13.08)	16 (13.22)	12 (12.90)	11 (7.59)					

ADHD, attention-deficit/hyperactivity disorder; SD, standard deviation; IQ = intelligence quotient.

To examine the association between comorbidities and treatment status we compared the three groups (drug-naïve ADHD vs. treated ADHD vs. controls) on comorbid disorders (Table 2). To further determine the effect of treatment duration on comorbidities, we compared the four groups: (1) drug-naïve ADHD; (2) treated ADHD ≦ one year (n = 36); (3) treated ADHD > one year (n = 57); (4) healthy controls (Table 3). We also compared comorbidities among childhood ADHD subtypes: (1) ADHD combined type (ADHD-C, n = 126); (2) ADHD inattentive type (ADHD-I, n = 86); (3) healthy controls (Table 4). Additionally, we conducted multivariate logistic regressions to identify significant predictors (e.g., sex, age onset of ADHD, medication history, ADHD subtypes, IQ for comorbid psychiatric diagnoses (see Table 5). Odds ratios (OR) and 95% confidence intervals (CI) were computed. To address the issue with multiple comparisons among subgroups, we adjusted alpha levels by using the Bonferroni correction method. We divided the alpha level (original alpha less than 0.05 level as significant) by the number of comparisons, i.e., 0.05/n, where n is the number of subgroup comparison. Despite the calculation and presentation of 95% CI in Tables 2, 3 and 4, we only interpreted the results that survived statistical significance with adjusted alpha levels after Bonferroni correction. For Table 2 and Table 4, the alpha level for significance is 0.05/3 = 0.017; for Table 3, the alpha level for significance is 0.05/6 = 0.008.

**Table 2:** Psychiatric comorbid conditions among drug-naïve adults with ADHD, adults with ADHD treated by medication, and controls.

Psychiatric diagnoses	Drug-naïve ADHD (n = 121)		Treated ADHD (n = 93)		Controls (n = 145)		Odds Ratio (95% CI) or p*		
							Drug-naïve ADHD vs. Controls	Treated ADHD vs. Controls	Drug-naïve ADHD vs. Treated ADHD
	N	(%)	N	(%)	N	(%)			
Oppositional defiant disorder	60	(49.59)	46	(49.46)	2	(1.38)	71.50 (16.93–301.99)	69.98 (16.36–299.39)	1.02 (0.59–1.76)
Conduct disorder	39	(32.23)	31	(33.33)	3	(2.07)	22.79 (6.83–76.09)	23.67 (6.97–80.33)	0.96 (0.54–1.71)
Tic Disorder	10	(8.26)	10	(10.75)	2	(1.38)	6.44 (1.38–29.99)	8.61 (1.84–40.26)	0.75 (0.30–1.88)
Anxiety disorders	46	(38.02)	27	(29.03)	4	(2.76)	21.62 (7.50–62.37)	14.42 (4.85–42.89)	1.50 (0.84–2.68)
Generalized anxiety disorder	23	(19.01)	5	(5.38)	0	(0)	< .001*	.005*	4.13 (1.51–11.33)
Specific phobia	20	(16.53)	9	(9.68)	3	(2.07)	9.37 (2.71–32.39)	5.07 (1.34–19.25)	1.85 (0.80–4.27)
Social phobia	21	(17.36)	10	(10.75)	0	(0)	< .001*	< .001*	1.74 (0.78–3.91)
Panic disorder	3	(2.48)	5	(5.38)	0	(0)	.057*	.005*	0.45 (0.10–1.92)
Obsessive compulsive disorder	3	(2.48)	4	(4.30)	1	(0.69)	3.66 (0.38–35.66)	6.47 (0.71–58.83)	0.57 (0.12–2.59)
Mood disorders	34	(28.10)	10	(10.75)	0	(0)	< .001*	< .001*	3.24 (1.51–6.98)
Dysthymic disorder	23	(19.01)	8	(8.60)	0	(0)	< .001*	< .001*	2.49 (1.06–5.87)
Major depression	10	(8.26)	2	(2.15)	0	(0)	< .001*	.076*	4.10 (0.88–19.18)
Bipolar disorder	1	(0.83)	0	(0)	0	(0)	.273*	—	.380*
Substance use disorders	16	(13.22)	11	(11.83)	0	(0)	< .001*	< .001*	1.12 (0.49–2.55)
Nicotine	16	(13.22)	11	(11.83)	0	(0)	< .001*	< .001*	1.12 (0.49–2.55)
Alcohol	5	(4.13)	2	(2.17)	0	(0)	.014*	.075*	1.94 (0.37–10.23)
Adjustment disorders	39	(32.23)	5	(5.38)	1	(0.69)	68.49 (9.24–507.77)	8.18 (0.94–71.18)	8.37 (3.15–22.27)
Sleep disorders	49	(40.50)	23	(24.73)	1	(0.69)	97.99 (13.26–723.99)	47.31 (6.26–357.46)	2.07 (1.14–3.75)
Eating disorders	6	(4.96)	5	(5.38)	0	(0)	.007*	.005*	0.93 (0.27–3.13)
Any psychiatric disorder	106	(87.60)	64	(68.82)	11	(7.59)	86.08 (37.96–195.17)	26.88 (12.63–57.21)	3.20 (1.60–6.42)

ADHD, attention-deficit/hyperactivity disorder; CI, confidence interval.

* Fisher exact *p* value.

**Table 3:** Psychiatric comorbid conditions among drug-naïve adults with ADHD, adults with ADHD treated for ≤ 1 year, adults with ADHD treated for > 1 year, and controls.

Psychiatric diagnoses N (%)	1. Drug-naïve ADHD (n = 121)	2. Treated ADHD < = 1year (n = 36)	3. Treated ADHD > 1year (n = 57)	4.Controls (n = 145)	Odds Ratio (95% CI) or p*					
					1 vs. 4	2 vs. 4	3 vs. 4	1 vs. 2	1 vs. 3	2 vs. 3
Oppositional defiant disorder	60 (49.59)	16 (44.44)	30 (52.63)	2 (1.38)	71.50 (16.93–301.99)	57.20 (12.23–267.52)	79.44 (17.92–352.24)	1.25 (0.59–2.64)	0.90 (0.48–1.69)	0.72 (0.31–1.67)
Conduct disorder	39 (32.23)	8 (22.22)	23 (40.35)	3 (2.07)	22.79 (6.83–76.09)	13.52 (3.38–54.15)	32.02 (9.08–112.88)	1.69 (0.70–4.04)	0.71 (0.37–1.37)	0.42 (0.16–1.09)
Tic Disorder	10 (8.26)	3 (8.33)	7 (12.28)	2 (1.38)	6.44 (1.38–29.99)	6.50 (1.04–40.46)	10.01 (2.01–49.77)	0.99 (0.26–3.81)	0.64 (0.23–1.79)	0.65 (0.16–2.69)
Anxiety disorders	46 (38.02)	13 (36.11)	14 (24.56)	4 (2.76)	21.62 (7.50–62.37)	19.92 (5.98–66.43)	11.48 (3.59–36.70)	1.09 (0.50–2.35)	1.88 (0.93–3.82)	1.74 (0.70–4.31)
Generalized anxiety disorder	23 (19.01)	3 (8.33)	2 (3.51)	0 (0)	< .001*	< .001*	.023*	2.58 (0.73–9.16)	6.45 (1.47–28.41)	2.50 (0.40–15.75)
Specific phobia	20 (16.53)	5 (13.89)	4 (7.02)	3 (2.07)	9.37 (2.71–32.39)	7.63 (1.73–33.64)	3.57 (0.77–16.49)	1.23 (0.43–3.54)	2.62 (0.85–8.07)	2.14 (0.53–8.56)
Social phobia	21 (17.36)	6 (16.67)	4 (7.02)	0 (0)	< .001*	< .001*	.001*	1.05 (0.39–2.84)	2.78 (0.91–8.53)	2.65 (0.69–10.14)
Panic disorder	3 (2.48)	1 (2.78)	4 (7.02)	0 (0)	.057*	.044*	.001*	0.89 (0.09–8.83)	0.34 (0.07–1.56)	0.38 (0.04–3.53)
Obsessive compulsive disorder	3 (2.48)	2 (5.56)	2 (3.51)	1 (0.69)	3.66 (0.38–35.66)	8.47 (0.75–96.16)	5.24 (0.47–58.91)	0.43 (0.07–2.69)	0.70 (0.11–4.31)	1.62 (0.22–12.03)
Mood disorders	34 (28.10)	8 (22.22)	2 (3.51)	0 (0)	< .001*	< .001*	.023*	1.37 (0.57–3.30)	10.75 (2.48–46.53)	7.86 (1.56–39.50)
Dysthymic disorder	23 (19.01)	7 (19.44)	1 (1.75)	0 (0)	< .001*	< .001*	.110*	0.97 (0.38–2.49)	13.14 (1.73–99.96)	13.52 (1.59–115.20)
Major depression	10 (8.26)	1 (2.78)	1 (1.75)	0 (0)	< .001*	.044*	.110*	3.15 (0.39–25.50)	5.05 (0.63–40.41)	1.60 (0.10–26.41)
Bipolar disorder	1 (0.83)	0 (0)	0 (0)	0 (0)	.273*	—	—	.584*	.491*	—
Substance use disorders	16 (13.22)	4 (11.11)	7 (12.28)	0 (0)	< .001*	< .001*	< .001*	1.18 (0.37–3.79)	1.09 (0.42–2.81)	0.92 (0.25–3.41)
Nicotine	16 (13.22)	4 (11.11)	7 (12.28)	0 (0)	< .001*	< .001*	< .001*	1.18 (0.37–3.79)	1.09 (0.42–2.81)	0.92 (0.25–3.41)
Alcohol	5 (4.13)	1 (2.78)	1 (1.75)	0 (0)	.014*	.044*	.110*	1.51 (0.17–13.35)	2.37 (0.27–20.78)	1.57 (0.10–25.95)
Adjustment disorders	39 (32.23)	2 (5.56)	3 (5.26)	1 (0.69)	68.49 (9.24–507.77)	8.47 (0.75–96.16)	8.00 (0.81–78.58)	8.09 (1.85–35.38)	8.56 (2.52–29.10)	1.06 (0.17–6.67)
Sleep disorders	49 (40.50)	11 (30.56)	12 (21.05)	1 (0.69)	97.99 (13.26–723.99)	63.36 (7.83–512.54)	38.40 (4.86–303.44)	1.55 (0.70–3.43)	2.55 (1.23–5.31)	1.65 (0.64–4.28)
Eating disorders	6 (4.96)	3 (8.33)	2 (3.51)	0 (0)	.007*	< .001*	.023*	0.58 (0.14–2.44)	1.45 (0.28–7.40)	2.50 (0.40–15.75)
Any psychiatric disorder	106 (87.60)	25 (69.44)	39 (68.42)	11 (7.59)	86.08 (37.96–195.17)	27.68 (10.83–70.76)	26.39 (11.50–60.56)	3.11 (1.28–7.59)	3.26 (1.50–7.10)	1.05 (0.43–2.59)

ADHD, attention-deficit/hyperactivity disorder; CI, confidence interval

* Fisher exact *p* value.

**Table 4:** Psychiatric comorbid conditions among childhood ADHD subtypes^a^.

Psychiatric diagnoses	Combined type (n = 126)		Inattention type (n = 86)		Controls (n = 145)		Odds Ratio (95% CI) or p*		
							Combined type vs. Controls	Inattention type vs. Controls	Combined type vs. Inattention type
	N	(%)	N	(%)	N	(%)			
Oppositional defiant disorder	81	(64.29)	24	(27.91)	2	(1.38)	131.63 (31.09–557.23)	27.68 (6.35–120.73)	4.76 (2.62–8.64)
Conduct disorder	60	(47.62)	9	(10.47)	3	(2.07)	43.69 (13.21–144.49)	5.53 (1.46–21.04)	7.90 (3.64–17.13)
Tic Disorder	17	(13.49)	3	(3.49)	2	(1.38)	11.15 (2.52–49.28)	2.58 (0.42–15.78)	4.32 (1.22–15.21)
Anxiety disorders	48	(38.10)	25	(29.07)	4	(2.76)	21.69 (7.54–62.41)	14.45 (4.82–43.29)	1.50 (0.83–2.70)
Generalized anxiety disorder	18	(14.29)	10	(11.63)	0	(0)	< .001*	< .001*	1.27 (0.55–2.90)
Specific phobia	20	(15.87)	9	(10.47)	3	(2.07)	8.93 (2.59–30.84)	5.53 (1.46–21.04)	1.61 (0.70–3.74)
Social phobia	20	(15.87)	11	(12.79)	0	(0)	< .001*	< .001*	1.29 (0.58–2.84)
Panic disorder	6	(4.76)	2	(2.33)	0	(0)	.008*	.065*	2.10 (0.41–10.66)
Obsessive compulsive disorder	7	(5.56)	0	(0)	1	(0.69)	8.47 (1.03–69.82)	.440*	.026*
Mood disorders	28	(22.22)	16	(18.60)	0	(0)	< .001*	< .001*	1.25 (0.63–2.48)
Dysthymic disorder	22	(17.46)	9	(10.47)	0	(0)	< .001*	< .001*	1.81 (0.79–4.15)
Major depression	5	(3.97)	7	(8.14)	0	(0)	.016*	< .001*	0.47 (0.14–1.52)
Bipolar disorder	1	(0.79)	0	(0)	0	(0)	.283*	—	.408*
Substance use disorders	20	(15.87)	7	(8.14)	0	(0)	< .001*	< .001*	2.15 (0.87–5.33)
Nicotine	20	(15.87)	7	(8.14)	0	(0)	< .001*	< .001*	2.15 (0.87–5.33)
Alcohol	7	(5.56)	0	(0)	0	(0)	.004*	—	.026*
Adjustment disorders	28	(22.22)	16	(18.60)	1	(0.69)	41.14 (5.51–307.40)	32.91 (4.28–253.22)	1.25 (0.63–2.48)
Sleep disorders	56	(44.44)	16	(18.60)	1	(0.69)	115.19 (15.62–849.29)	32.91 (4.28–253.18)	3.50 (1.83–6.68)
Eating disorders	9	(7.14)	2	(2.33)	0	(0)	.001*	.065*	3.26 (0.69–15.47)
Any psychiatric disorder	114	(90.48)	54	(62.79)	11	(7.59)	115.71 (49.19–272.19)	20.56 (9.67–43.70)	5.63 (2.69–11.78)

ADHD, attention-deficit/hyperactivity disorder; CI, confidence interval

^a^ two cases of ADHD, hyperactivity-impulsivity subtype, were excluded from the analysis

* Fisher exact *p* value.

**Table 5:** Multivariate analysis.

Odds Ratio (95% CI)	Oppositional defiant disorder	Conduct disorder	Anxiety disorders	Mood disorders	Sleep disorders
Sex	1.39 (0.71–2.71)	1.22 (0.57–2.60)	0.52 (0.27–1.01)	0.90 (0.42–1.91)	0.90 (0.45–1.79)
Age of onset	0.92 (0.78–1.09)	0.91 (0.75–1.12)	0.96 (0.84–1.09)	1.04 (0.90–1.19)	1.04 (0.93–1.16)
Age of treatment	1.00 (0.95–1.04)	0.97 (0.92–1.02)	1.00 (0.95–1.04)	1.01 (0.96–1.07)	1.00 (0.95–1.05)
Medication history					
Ever use	1.33 (0.47–3.72)	0.73 (0.24–2.19)	0.43 (0.14–1.32)	2.35 (0.64–8.64)	0.73 (0.26–2.08)
Current use	0.64 (0.26–1.56)	0.67 (0.26–1.69)	3.39 (1.22–9.41)*	0.33 (0.09–1.16)	1.07 (0.43–2.66)
Duration of use	1.00 (0.99–1.01)	1.00 (0.99–1.01)	0.99 (0.98–1.01)	0.94 (0.90–0.99)*	0.99 (0.98–1.00)*
Childhood ADHD subtypes					
Combined subtype	4.10 (2.14–7.85)*	7.40 (3.21–17.10)*	1.68 (0.88–3.23)	1.53 (0.72–3.24)	4.32 (2.14–8.69)*
Full-scale IQ	0.97 (0.94–1.00)	0.98 (0.95–1.01)	1.00 (0.97–1.03)	0.99 (0.96–1.03)	0.99 (0.96–1.02)

ADHD, attention-deficit/hyperactivity disorder; CI, confidence interval

*p < 0.05

## Results

### Sample characteristics

Table 1 presents results of the comparisons between the ADHD and controls, as well as among the three groups. The three-group comparison revealed that drug-naïve

ADHD and controls had higher Full-scale IQ, Performance IQ, Verbal IQ (control only) and years of education than treated ADHD. Males were more predominant in treated ADHD than in drug-naïve ADHD and controls. Drug-naïve ADHD had more severe inattentive and hyperactive-impulsive (adulthood only) symptoms during childhood and adulthood than treated ADHD. Because of the between-group differences in age, sex, educational years, and Full-scale IQ, we controlled for these variables in our subsequent analyses.

### Psychiatric comorbid conditions by treatment status

Table 2 displays current psychiatric comorbid conditions for drug-naïve ADHD, treated ADHD, and controls. Drug-naïve ADHD and treated ADHD were more likely than controls to have psychiatric disorders including ODD, CD, tic disorder, anxiety disorders (including GAD, specific phobia, social phobia), mood disorders such as dysthymic disorder, substance use disorders (e.g., nicotine use disorder), sleep disorders, and eating disorders. Also, drug-naïve ADHD had a higher likelihood of having MDD, alcohol use disorder, and adjustment disorder than controls. Panic disorder was more prevalent in treated ADHD than controls. Drug-naïve ADHD, compared to treated ADHD, were more likely to have GAD, mood disorders, adjustment disorder, and sleep disorders.

### Comorbid psychiatric conditions by treatment duration

Table 3 displays comorbid psychiatric conditions among drug-naïve ADHD, treated ADHD with medication for one year and less (ADHD-TS), treated ADHD with medication longer than one year (ADHD-TL), and controls. Among ADHD groups specifically, drug-naïve ADHD had a higher likelihood of having adjustment disorders than ADHD-TS. Drug-naïve ADHD was more likely than ADHD-TL to have psychiatric disorders including GAD, mood disorders, and adjustment disorders. There were no between-group differences in OCD or bipolar disorders across the ADHD groups and controls.

### Psychiatric comorbid conditions across different childhood ADHD subtypes

Participants with childhood ADHD-C were more likely to have tic disorder, panic disorder, and alcohol use disorder than those with childhood ADHD-I and controls. Moreover, participants with childhood ADHD-C were more likely to have eating disorder than controls and more likely to have ODD, CD, and sleep disorders than childhood ADHD-I (Table 4).

The psychiatric comorbid patterns across different ADHD subtypes at the current assessment (adult ADHD subtypes) are presented in S1 Table. In general, both ADHD subtypes were more likely to have all psychiatric comorbid conditions assessed herein than controls except bipolar disorder. Adults with ADHD-C adults were more likely to have lifetime ODD, CD, and sleep disorders than adults with ADHD-I.

### Associated factors of comorbid psychiatric disorders

Multivariate analyses revealed that childhood ADHD-C was associated with a higher risk of ODD, CD, and sleep disorders. Current use of medication was associated with an higher risk of anxiety disorders. Longer duration of medication use was associated with lower risks of mood disorders and sleep disorders (Table 5). Univariate analyses demonstrated similar results (S2 Table).

## Discussion

The current study is one of the few studies that examined comprehensively the psychiatric comorbid patterns in adults with ADHD with regards to the effects of treatment history and duration, as well as childhood ADHD subtypes. We found that adults with ADHD, in general, had significantly more comorbid psychiatric conditions than controls. Drug-naïve adults with ADHD, relative to treated ADHD and controls, had more core ADHD symptoms during childhood and adulthood (except for childhood hyperactivity/impulsivity) and more psychiatric comorbidities, particularly GAD, mood disorders, adjustment disorders, and sleep disorders. Childhood ADHD-C subtype, relative to ADHD-I, was associated with higher risks of ODD, CD, OCD, alcohol use disorder, and sleep disorders. Importantly, we found that childhood ADHD-C was a strong correlate of several common comorbid conditions including ODD, CD, and sleep disorders. Current use of medication and a shorter duration of medication use were associated with anxiety and mood disorders, respectively.

Similar to our adolescent ADHD study [20] and previous adult ADHD studies [7], we found that adults with ADHD were more likely to have comorbid psychiatric conditions. High rates of comorbid conditions were present in both drug-naïve ADHD or treated ADHD, regardless of ADHD subtypes and comorbid conditions such as ODD [4, 10], CD [4, 75], tic disorder [4], anxiety disorders [4, 9], GAD [3, 4, 9], specific phobia [4, 9], social phobia [4, 8, 9], mood disorders [4, 8, 9], dysthymic disorder [9, 51], substance use disorders [3, 8, 9, 55, 75], nicotine use disorder [75], sleep disorders [56], and eating disorders [4, 55]. Moreover, our findings of only drug-naïve adult ADHD but not treated adult ADHD having increased risks for MDD and alcohol use disorder provide evidence to support that treatment with medication may offset the occurrence of MDD [3, 4, 8, 9, 11, 55] and alcohol use disorder [8, 51].

No studies have examined the association between treatment effects and panic disorder or specific phobia in adults with ADHD. Our result contributes to the literature by showing that panic disorder was more prevalent in treated ADHD adults than controls. Whether this finding is due to treatment-induced panic-like side effects requires further clarification from future research. Another novel finding is that specific phobia was more prevalent in drug-naïve ADHD and ADHD-TS than controls, suggesting that long-term treatment (> one year), relative to no treatment or short-term treatment (≤ one year), was associated with a decreased risk of specific phobia in adults with ADHD. A prospective follow-up study of these patients is warranted to test the causal effect i.e., whether longer treatment duration indeed decreases the risk of specific phobia.

Previous studies reported that patients with ADHD treated for at least one year did not differ from drug-naïve ADHD in the risk of MDD [33] and that adults with ADHD treated for more than two years had better functioning (self-reported improvement) than those treated for less than two years [76]. These results suggest that pharmacotherapy for ADHD may be most beneficial in reducing comorbidities if it is longer than one year [33], even two years [76]. In the current study, we did not find any differences in comorbidities between those with ADHD who received treatment for longer than one year vs. shorter than one year. However, we found that adults with drug-naïve ADHD were more likely to have sleep disorders than treated ADHD and longer treatment reduced the risk for sleep disorders. This is supported by some studies suggesting that treatment reduces the risk of sleep disorders in ADHD [19, 77]. This finding might be explained by the improvement in life quality with medication treatment, which subsequently reduces the risk of sleep disorders, as evidenced in our prior clinical trial [13].

Our finding that current use of medication increases the risk of anxiety disorder is contradictory to the studies showing that treatment decreased [22, 23, 78] or did not increase the risk of anxiety symptoms [28]. Possible explanations are that medication may cause anxiety symptoms to be diagnosed as anxiety disorders [13]; or that the insufficient response to medication among patients with ADHD comorbid with anxiety may contribute to increased likelihood of current use of medications [79]. These possibilities should be examined in future research with a larger sample of ADHD with or without current medication. Additionally, it is imperative to assess anxiety symptoms and disorders among adults with ADHD who are currently taking medication for treating ADHD. Moreover, the association between shorter duration of treatment and an increased risk of mood disorders is in line with previous research reporting that no treatment or shorter treatment duration increased the risk of substance use [17], which then predicted later risks of mood disorders [11].

Our findings strongly support more instances of psychiatric disorders in adults with ADHD-C than adults with ADHD-I [6, 54]. Specifically, consistent with previous studies, we found that ODD [36, 38, 51, 80], CD [6, 36–39, 52, 53], alcohol use disorder [45], and sleep disorders [17, 47, 48] were more prevalent in adults with childhood ADHD-C than adults with childhood ADHD-I. Our previous research in adolescents has provided strong evidence of high comorbidities of ODD and CD in ADHD-C [20]. Hence, psychiatric comorbid patterns also contribute to the differential diagnosis of these two distinct ADHD subtypes. These findings suggest that more clinical and research attention should be devoted to young adults with childhood ADHD-C, as they are more likely to have disruptive behaviors and disorders, which are highly associated with antisocial behaviors [6, 51], substance use disorders [69], and criminality [51, 81].

### Strengths and limitations

This study is the first one to examine a wide range of psychiatric comorbidities by using (semi-)structured diagnostic interviews rather than self-reported questionnaires among adults with ADHD and to investigate comprehensively the effects of treatment and treatment duration, as well as childhood ADHD subtypes, on the psychiatric comorbid patterns. Despite this, several limitations merit comments. First, the directionality and causality of the significant findings remain to be determined. Second, although our longitudinal pilot study showed a high correlation between adolescent/mother reports and adult self-reports about the participants’ ADHD symptoms [71], recall bias may still exist. Third, as for treatment effects, this study is not a randomized controlled trial, and treatment in observational studies is usually confounded by indications. Although adults with severe symptoms and impairments are more likely to be treated with medication, which may draw the comparison to the null, we still find some significant results. Lastly, this study may suffer from selection bias during sample ascertainment because we did not recruit the sample from a representative epidemiological sample. Instead, ADHD adults were recruited from the hospitals through physicians’ referrals and the community through advertisement.

## Conclusion

Our findings suggest that ADHD often persists into adulthood and is still associated with high psychiatric comorbidity. Treatment with ADHD medications may reduce comorbidity. Moreover, childhood ADHD-C is associated with a greater risk of adult comorbidities than ADHD-I and is one of the significant correlates of adult comorbid conditions. The findings suggest that for adult ADHD, medication treatment should be considered as one of the necessary treatment modalities to prevent the development of psychiatric comorbid conditions. For children with ADHD-C, medication treatment may likewise be helpful to minimize the risks of adverse psychiatric outcomes in adulthood, decreasing social impairment and improving life quality [71].

## Supporting information

S1 Table Psychiatric comorbid conditions among ADHD subtypes of adulthood. (DOC)

S2 Table Univariate analysis. (DOC)
