# Supplementary material for: Psychiatric comorbid patterns in adults with attention-deficit hyperactivity disorder: Treatment effect and subtypes
Source: PLoS One. 2019 Feb 7;14(2):e0211873. doi: 10.1371/journal.pone.0211873 (PMC6366698; doi:10.1371/journal.pone.0211873)
Supplement: S1 Table — (DOC) [file pone.0211873.s001.doc]

**S1 Table**. Psychiatric comorbid conditions among ADHD subtypes of adulthood

| Psychiatric diagnoses | Combined type  (n=79) | | Inattention type (n=101) | | Control  (n=145) | |  | Odds Ratio (95% CI) or *p** | | |
| --- | --- | --- | --- | --- | --- | --- | --- | --- | --- | --- |
|  |  |  |  |  |  |  |  | Combined type vs. Control | Inattention type vs. Control | Combined type vs. Inattention type |
|  | N | (%) | N | (%) | N | (%) |  |  |  |  |
| Oppositional defiant disorder | 48 | (60.76) | 42 | (41.58) | 2 | (1.38) |  | 114.40 (26.35-496.67) | 50.90 (11.93-217.11) | 2.25 (1.23-4.11) |
| Conduct disorder | 37 | (46.84) | 21 | (20.79) | 3 | (2.07) |  | 42.72 (12.53-145.67) | 12.43 (3.59-42.95) | 3.44 (1.79-6.62) |
| Tic Disorder | 7 | (8.86) | 8 | (7.92) | 2 | (1.38) |  | 6.95 (1.41-34.32) | 6.15 (1.28-29.60) | 1.13 (0.39-3.26) |
| Anxiety disorders | 31 | (39.24) | 36 | (35.64) | 4 | (2.76) |  | 22.77 (7.64-67.82) | 19.52 (6.67-57.14) | 1.17 (0.64-2.14) |
| Generalized anxiety disorder | 16 | (20.25) | 11 | (10.89) | 0 | (0) |  | <.001* | <.001* | 2.08 (0.90-4.78) |
| Specific phobia | 11 | (13.92) | 15 | (14.85) | 3 | (2.07) |  | 7.66 (2.07-28.34) | 8.26 (2.32-29.34) | 0.93 (0.40-2.15) |
| Social phobia | 13 | (16.46) | 17 | (16.83) | 0 | (0) |  | <.001* | <.001* | 0.97 (0.44-2.15) |
| Panic disorder | 4 | (5.06) | 4 | (3.96) | 0 | (0) |  | .006* | .016* | 1.29 (0.31-5.34) |
| Obsessive compulsive disorder | 5 | (6.33) | 1 | (0.99) | 1 | (0.69) |  | 9.73 (1.12-84.83) | 1.44 (0.09-23.29) | 6.76 (0.77-59.07) |
| Mood disorders | 23 | (29.11) | 19 | (18.81) | 0 | (0) |  | <.001* | <.001* | 1.77 (0.88-3.56) |
| Dysthymic disorder | 17 | (21.52) | 12 | (11.88) | 0 | (0) |  | <.001* | <.001* | 2.03 (0.91-4.56) |
| Major depression | 5 | (6.33) | 7 | (6.93) | 0 | (0) |  | .002* | .001* | 0.91 (0.28-2.98) |
| Bipolar disorder | 1 | (1.27) | 0 | (0) | 0 | (0) |  | .175* | -- | .257* |
| Substance use disorders | 12 | (15.19) | 13 | (12.87) | 0 | (0) |  | <.001* | <.001* | 1.23 (0.53-2.87) |
| Nicotine | 12 | (15.19) | 13 | (12.87) | 0 | (0) |  | <.001* | <.001* | 1.23 (0.53-2.87) |
| Alcohol | 4 | (5.06) | 2 | (1.98) | 0 | (0) |  | .006* | .089* | 2.68 (0.48-15.00) |
| Adjustment disorders | 21 | (26.58) | 21 | (20.79) | 1 | (0.69) |  | 52.14 (6.85-396.63) | 37.80 (4.99-286.27) | 1.38 (0.69-2.76) |
| Sleep disorders | 36 | (45.57) | 31 | (30.69) | 1 | (0.69) |  | 120.55 (16.06-904.99) | 63.77 (8.53-476.66) | 1.89 (1.03-3.49) |
| Eating disorders | 7 | (8.86) | 3 | (2.97) | 0 | (0) |  | <.001* | .037* | 3.22 (0.81-12.89) |
| Any psychiatric disorder | 71 | (89.87) | 80 | (79.21) | 11 | (7.59) |  | 108.10 (41.60-280.94) | 46.40 (21.27-101.25) | 2.33 (0.97-5.59) |

ADHD, attention deficit/hyperactivity disorder; CI, confidence interval.

* Fisher exact *p* value.
