# Supplementary material for: Psychiatric comorbid patterns in adults with attention-deficit hyperactivity disorder: Treatment effect and subtypes
Source: PLoS One. 2019 Feb 7;14(2):e0211873. doi: 10.1371/journal.pone.0211873 (PMC6366698; doi:10.1371/journal.pone.0211873)
Supplement: S2 Table — (DOC) [file pone.0211873.s002.doc]

**S2 Table.** Univariate analysis

|  | Oppositional defiant disorder | | Conduct disorder | | Anxiety disorders | | Mood disorders | | Sleep disorders | |
| --- | --- | --- | --- | --- | --- | --- | --- | --- | --- | --- |
|  | *p* | OR (95% CI) | *p* | OR (95% CI) | *p* | OR (95% CI) | *p* | OR (95% CI) | *p* | OR (95% CI) |
| Sex | .092 | 1.64 (0.92-2.92) | .088 | 1.74 (0.92-3.28) | .038 | 0.54 (0.30-0.97) | .172 | 0.62 (0.32-1.23) | .713 | 0.89 (0.49-1.62) |
| Age of onset | .082 | 0.87 (0.74-1.02) | .025 | 0.81 (0.68-0.98) | .375 | 0.94 (0.81-1.08) | .974 | 1.00 (0.88-1.13) | .696 | 0.98 (0.87-1.10) |
| Age of treatment | .272 | 0.98 (0.95-1.01) | .031 | 0.97 (0.93-1.00) | .062 | 1.03 (1.00-1.07) | .007 | 1.06 (1.02-1.10) | .208 | 1.02 (0.99-1.05) |
| Medication history |  |  |  |  |  |  |  |  |  |  |
| Ever use | .592 | 1.17 (0.66-2.08) | .973 | 0.99 (0.54-1.82) | .975 | 1.01 (0.55-1.85) | .051 | 0.51 (0.26-1.00) | .321 | 0.74 (0.41-1.34) |
| Current use | .536 | 0.84 (0.49-1.45) | .204 | 0.69 (0.39-1.23) | .049 | 1.78 (1.00-3.16) | .107 | 0.57 (0.29-1.13) | .630 | 0.87 (0.49-1.54) |
| Duration of use | .402 | 1.00 (1.00-1.01) | .053 | 1.01 (1.00-1.02) | .095 | 0.99 (0.98-1.00) | .006 | 0.95 (0.92-0.99) | .115 | 0.99 (0.98-1.00) |
| Dosage/per day | .954 | 1.00 (0.97-1.03) | .386 | 1.02 (0.98-1.05) | .150 | 1.03 (0.99-1.06) | .023 | 0.94 (0.88-0.99) | .476 | 0.99 (0.95-1.02) |
| ADHD subtypes |  |  |  |  |  |  |  |  |  |  |
| Combined type | <.001 | 4.76 (2.62-8.64) | <.001 | 7.90 (3.64-17.13) | .176 | 1.50 (0.83-2.70) | .524 | 1.25 (0.63-2.48) | <.001 | 3.50 (1.83-6.68) |
| Full-scale IQ | .213 | 0.98 (0.96-1.01) | .670 | 0.99 (0.97-1.02) | .771 | 1.00 (0.97-1.02) | .478 | 1.01 (0.98-1.05) | .793 | 1.00 (0.98-1.03) |

ADHD, attention deficit/hyperactivity disorder; CI, confidence interval; OR, Odds Ratio.
